# Oxidation behavior and performance deterioration of cracking furnace tubes in service

**DOI:** 10.1016/j.heliyon.2024.e24500

**Published:** 2024-01-24

**Authors:** Chenyang Du, Ce Song, Xiaowei Li, Jun Yuan, Chang Liu, Yanchao Xin, Jianyu Lu

**Affiliations:** China Special Equipment Inspection and Research Institute, Beijing 100029, China

**Keywords:** Pyrolysis furnace tube, Cr35Ni45Nb, Oxidation, Endurance lifetime

## Abstract

In recent years, the centrifugal casting material Cr35Ni45Nb has been widely used in cracking furnace tubes. The common failure forms in the service process are carburizing cracking, bending, bulging, creep cracking, thermal fatigue cracking, thermal shock cracking, and oxidation, among which the inner wall oxidation and carburization of cracking furnace tubes cause the largest proportion of material failure. In this paper, we will discuss the inner wall oxidation behavior of cracking furnace tubes and its influence on the lasting strength of the furnace tubes. Several groups of endurance tests were designed for service furnace tubes, and the oxidation characteristics, oxide film rupture damage, and its influence on the endurance life of furnace tubes in different service times were analyzed by means of XRD, SEM, and so on. The results show that the oxide layer of the furnace tube is divided into two layers, the outer layer is repeatedly destroyed and rebuilt. With the continuous evolution of material structure, its properties also deteriorate, and its tensile strength, yield strength, elongation, and durable life all decrease significantly.

## 1 Introduction

The cracking furnace tube is the core component of the cracking furnace, and its service environment is very harsh and its working temperature is high. The tube wall of the furnace tube is in the environment of hydrocarbon carburization, oxidation, and vulcanization inside and outside the tube and high temperature, and at the same time, it is subjected to complex stress actions such as internal pressure, self-weight, temperature difference, fatigue and thermal shock caused by start-up and shutdown [[1], [2], [3]]. The common failure forms of ethylene cracking furnace tubes are carburizing cracking, bending, bulging, creep cracking, thermal fatigue cracking, thermal shock cracking, and oxidation, among which the failure rate of furnace tubes is the largest due to oxidation and carburization of the inner wall of furnace tubes [[4], [5], [6], [7]]. At present, researchers have rich research on the carburization of furnace tubes, but relatively little research on oxidation. Wang Hui et al. [8] found that there is a process of mutual promotion between carburization and oxidation of the Cr35Ni45 steel furnace tube through the study of its oxidation mechanism in long-term service. Du Chen-yang et al. [9,10] studied the detection method of carburization damage of cracking furnace tube based on a magnetic analyzer, ultrasonic guided wave, low-frequency electromagnetism, and other technical means, which provides a new method and means for the practical engineering application of carburization damage of cracking furnace tube. Stamatis A. Sarris [11] and others studied the effect of oxidation on the coking of furnace tubes and found that the oxide of Cr can play an anti-coking role. He Wen-long [12] studied the oxidation kinetics weight gain and oxidation kinetics thickening behavior of Cr25Ni35 steel at different oxidation temperatures and times through high-temperature oxidation experiments with and without stress. The results show that external load can affect the oxide layer and surface morphology of the furnace tube, and the cracking and peeling of the oxide film can be weakened under appropriate thermometer tensile stress. Timotijević, Milica [13]. Analyzed the fracture surface of an HP40Nb alloy furnace tube which failed after 11 years of service, and tested the mechanical properties of the failed parts. The results showed that the mechanical properties of the failed parts decreased greatly, and the brittle intergranular fracture was caused by a large number of precipitated and coarsened intergranular carbides at the grain boundary. Cheng-ming Fuyang [14] carried out failure treatment and mechanical tests on the aged HP40Nb alloy. The experimental results show that the interdendritic precipitates are continuously agglomerated and coarsened during aging treatment. With the decrease of exposure, the tensile strength and microhardness increased slightly, while the elongation and impact toughness decreased. The evolution of microstructure during aging is consistent with the change in mechanical properties. At present, there is no relevant research on the oxidation behavior of the furnace tube after service and its influence on the high-temperature durable life.

In this paper, the furnace tubes with different service times are taken as the research object, and the inner wall oxidation behavior of cracking furnace tubes during service is discussed emphatically. Twenty-four groups of endurance tests were carried out on new furnace tubes to observe the formation and rupture damage of oxide film, and to study the oxidation mechanism and its influence mechanism on high-temperature endurance life.

## 2 Experimental materials and methods

The experimental material is a Cr35Ni45 radiant section furnace tube cut from the ethylene cracking unit of a petrochemical company. The actual operating temperature is 1080 °C, and the service time is the new furnace tube, 2 years and 6 years respectively. The furnace tube is made by centrifugal casting, and the chemical composition of the original as-cast furnace tube material is shown in Table 1.

**Table 1:** Chemical composition of as-cast furnace tubes.

Element	C	Nb	Cr	Ni	Ti	Si	Fe
Percentage/(wt,%)	0.5	1.0	35.44	43.57	0.01	1.6	17.88

The arc-shaped block samples with the size of 10×15×7mm cut out on each of the above three furnace tubes are numbered A, B, and C from short to long according to the service life. After grinding and polishing, the sample was etched by electrolysis for 5 s at a voltage of 5 V, and the electrolyte ratio was ${150\text{ml}\;H}_{3}PO_{4}+10\text{ml}\;H_{2}SO_{4}+15g\;\text{Cr}O_{3}$ Furthermore, the morphology and phase composition were observed by 9XB-PC optical microscope, JSM-6510A scanning electron microscope, and electron probe respectively.

In addition, a length of 20 mm was cut off from the furnace tube that had been in service for 2 years, and the oxide layers on the inner wall and the outer wall were polished off. The extract was extracted for 15 h at a DC voltage of 5 V, and the composition of the extract was ${10\text{ml}\;\text{HCl}+90\%\;\text{CH}}_{3}\text{OH}$ (volume ratio). XRD analysis of the extracted products was carried out by using a Rigaku D/Max-Rb diffractometer.

The tensile properties of furnace tubes at room temperature were tested by MTS 810 material testing machine. High-temperature endurance test of the furnace tube was carried out with the UHRD304-B1 testing machine.

## 3 Experimental results

### 3.1 Oxidation behavior of furnace tubes in service

**Fig. 1:**
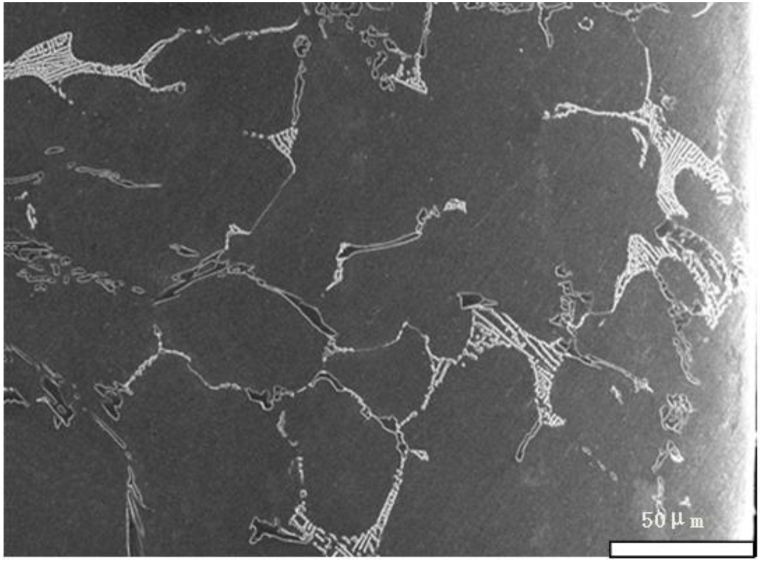
Organization of the inner wall of the unused furnace tube.

**Fig. 2:**
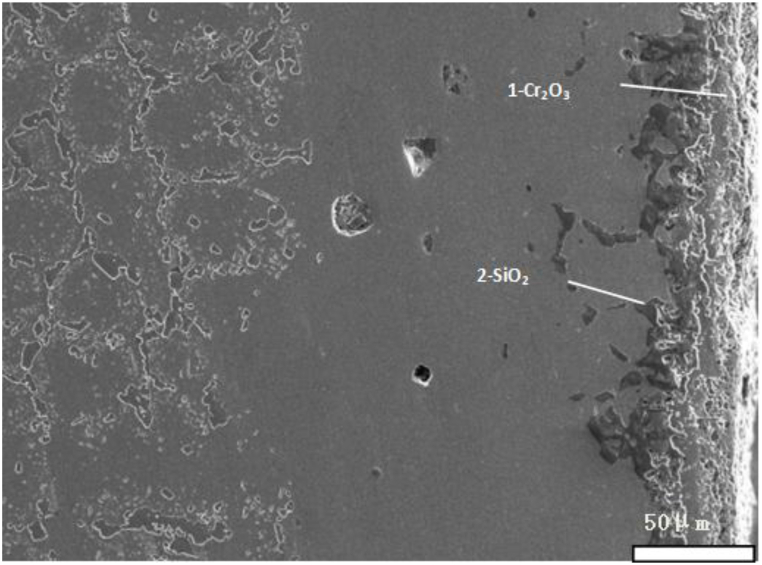
Organization of the inner wall of the furnace tube after 2 years of service.

**Fig. 3:**
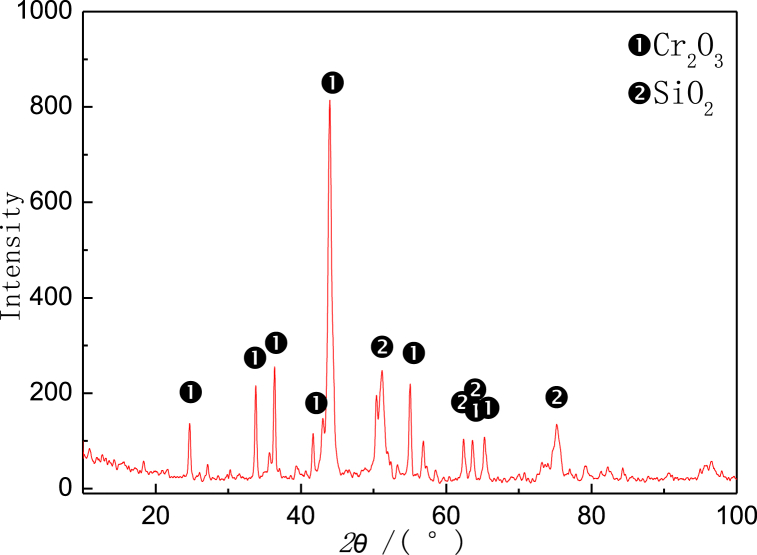
Composition of an oxide film.

**Fig. 4:**
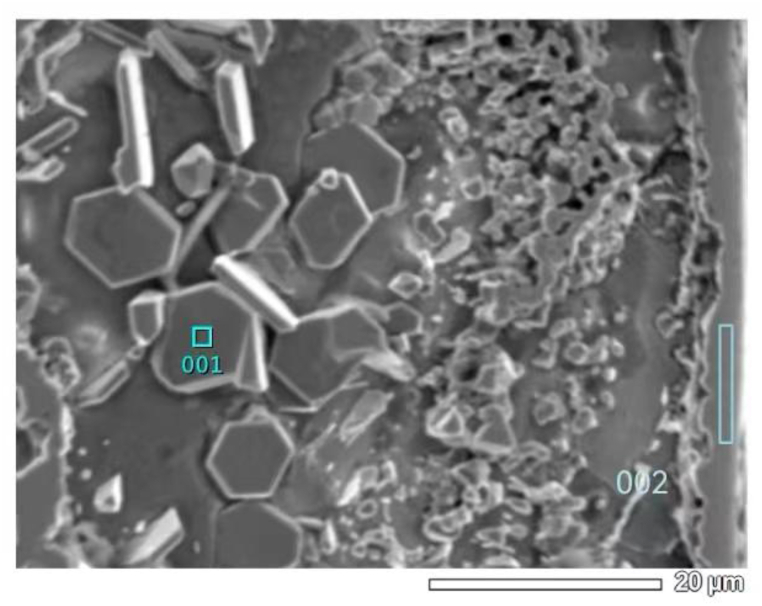
Electron probe micrograph.

We take new furnace tubes, furnace tubes that have been in service for 2 years, and furnace tubes that have been in service for 6 years as the research objects, and observe their microstructure characteristics through SEM. Fig. 1 shows the inner wall structure of a new furnace tube, which can be seen to be composed of strip eutectic carbide M_23_C_6_ and network NbC. Fig. 2 shows the inner wall structure of the furnace tube after two years of service. It can be seen that the structure of the furnace tube is relatively new, and the network NbC structure disappears and an obvious oxide layer appears. The XRD diffraction analysis (see Fig. 3) and electron probe analysis (see Table 2 and Fig. 4) show that the composition of the oxide layer is not single, but divided into two layers, the outer layer is a dense continuous film-like Cr_2_O_3_, and the inner layer is a discontinuous SiO_2_.

**Table 2:** Results of fixed-point analysis by electron probe.

Element/atom%		C	Si	Cr	Ni	Fe	Nb	O	Phase
position	1	0.88	0.03	34.97	0.26	0.74	0.08	63.04	Cr_2_O_3_
	2	0.19	28.14	0.17	0.16	0.13	0.00	71.21	SiO_2_
	3	0.08	0.03	31.55	0.08	0.55	0.00	67.71	Cr_2_O_3_
	4	0.48	26.16	0.11	0.11	0.07	0.00	73.07	SiO_2_

**Fig. 5:**
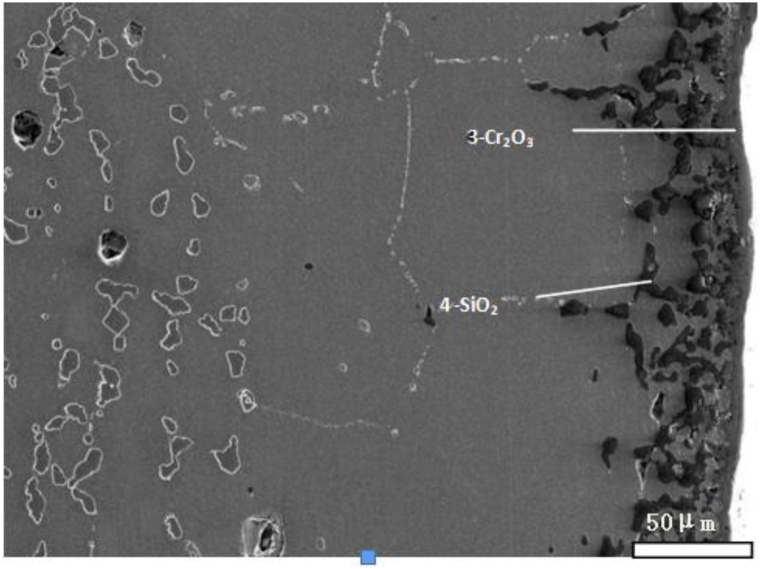
Organization of the inner wall of the furnace tube after 6 years of service.

Fig. 5 shows the organization of the inner wall after 6 years of service. The initial net-like NbC organization disappeared and an obvious oxide layer appeared, similar to that in the 2-year-old furnace tube and is divided into two internal and external layers. The thickness of the external oxide layers decreased, likely because of scorching or thermal shock [15,16], and the destruction of the external oxide layer decreased the protective effects on the furnace tube.

**Fig. 6:**
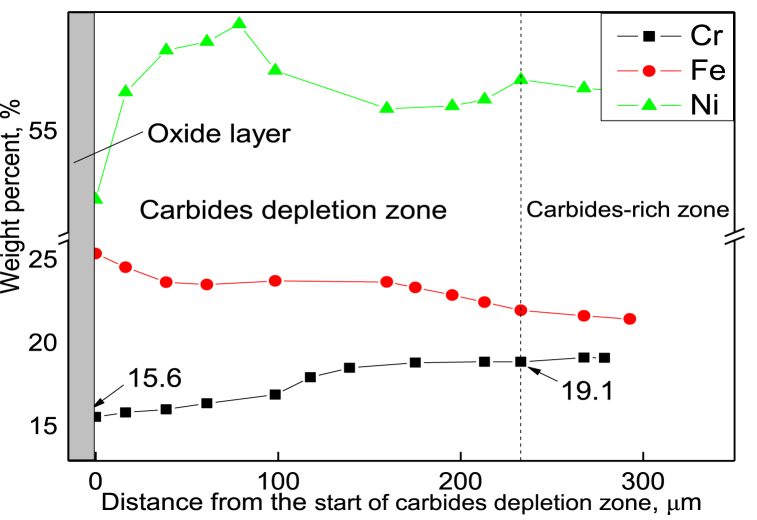
Changes in elemental concentrations in the depleted zone of the furnace tube material organization after 6 years of service.

**Fig. 7:**
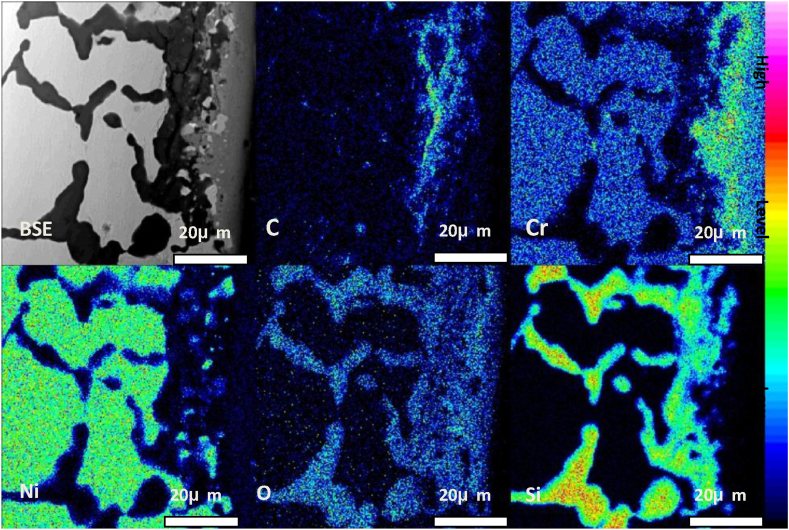
Element distribution in the inner wall area of furnace tube after six years of service.

A comparison between the organizations of the inner wall displayed in Fig. 2, Fig. 5 revealed that the width of the depleted zone of the 6-year service furnace tube was larger than that of the 2-year service furnace tube. Fig. 6 shows the concentration distribution curve of each element in the carbide-depleted zone of the 6-year-old furnace tube, indicating that the concentration of Cr in the depleted zone decreased with the decrease in the distance from the oxide film. The lowest concentration of Cr near the surface oxide layer was only approximately 15.6 %; at the end of the carbide-depleted zone of the Cr35Ni45Nb alloy, the Cr content was approximately 19.0 % (see Fig. 7), which facilitates carbide precipitation in the carbide-depleted zone. This indicates that 19.0 % was the critical concentration for carbide precipitation in the carbide-depleted zone of the Cr35Ni45Nb alloy, and the carbide could exist stably without decomposition for Cr concentrations higher than this value, resulting in the formation of an internal carbide enrichment zone.

**Fig. 8:**
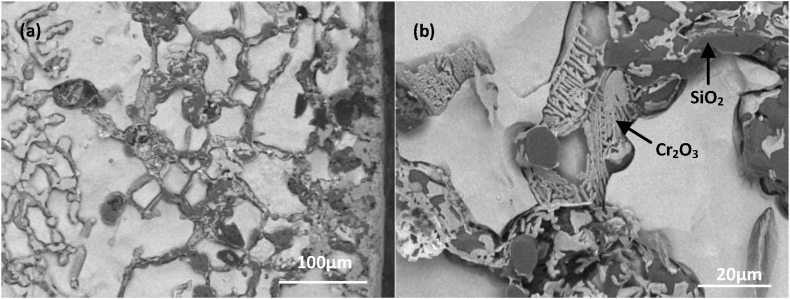
Carbide oxidation pattern on the inner side of a tube with deep leaching after 6 years of service.

The organization of the furnace tube after 6 years of service was not uniform at each location. Fig. 8 illustrates the deep erosion organization of the inner side of the furnace tube at another location. Large amounts of oxides were distributed at the grain boundaries of the original carbide-depleted region. According to Wagner's theory [[17], [18], [19], [20], [21]] and the oxide film rupture mechanism proposed by Evans [22], the nucleation of both Cr_2_O_3_ and SiO_2_ first occurred perpendicular to the metal matrix [23,24]. We divided the furnace tube tissue degradation process into five stages: (1) formation of the initial surface oxide layer, (2) oxidation of the carbon-containing atmosphere, (3) redistribution of Cr and C within the tubes, (4) internal carburization, and (5) oxidation of carbides. Fig. 4 displays the organization in stage (3), while Fig. 6(a) displays the organization of the furnace tube after 6 years of service that is in stage (5). Thus, the furnace tube after 6 years of service can be considered to have transitioned from stage (3) to (5). During this transition, the local oxide film lost resistance to the infiltration of O and C when the subsurface concentration of Cr could no longer promote the continuity of the surface oxide film. Therefore, large amounts of C and O infiltrated to the interior of the alloy. Moreover, the internal carbonization of the alloy (stage (4)) occurred first because the diffusion rate of C was considerably higher than that of O, and a large amount of relatively stable M_7_C_3_ carbide with high carbon activity precipitated and polymer coarsening occurred in the carbide-depleted region. Subsequently, with the gradual infiltration of O, the generated M_7_C_3_ was gradually oxidized, and the lower partial pressure of O in the alloy relative to the vicinity of the inner wall of the furnace tube promoted the competitive growth of Si and Cr [23,24]. The growth of SiO_2_ was dominant, such that SiO_2_ was formed preferentially, and when the SiO_2_ precipitation reaches saturation, an interface was gradually generated between SiO_2_ and the Cr_2_O_3_ substrate (stage (5); Fig. 8 (b)).

**Fig. 9:**
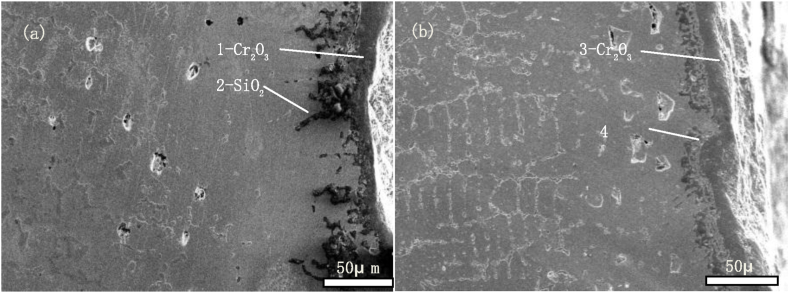
Organization of the outer wall of the furnace tube after (a) 2 years of service; (b) 6 years of service.

The tissue structure distribution of the outer wall of the furnace tube (Fig. 9) was more similar to that of the inner wall, which also had a composite oxide layer composed of Cr_2_O_3_ and SiO_2_ and a carbide-depleted zone, but no carbide-rich zone. The radiation section of the furnace tube was heated using radiation. The outer wall near the oxidizing environment was not exposed to any carburizing atmosphere, whereas the inner wall of the furnace tube underwent cracking reaction and was exposed to an oxidizing–carburizing atmosphere.

### 3.2 Mechanical properties of furnace tubes after different service periods at room temperature

The mechanical properties of an unused furnace tube, a tube in service for 2 years, and a tube in service for 6 years were evaluated at room temperature; the results were analyzed.

#### 3.2.1 Mechanical property test of the furnace tube

**Fig. 10:**
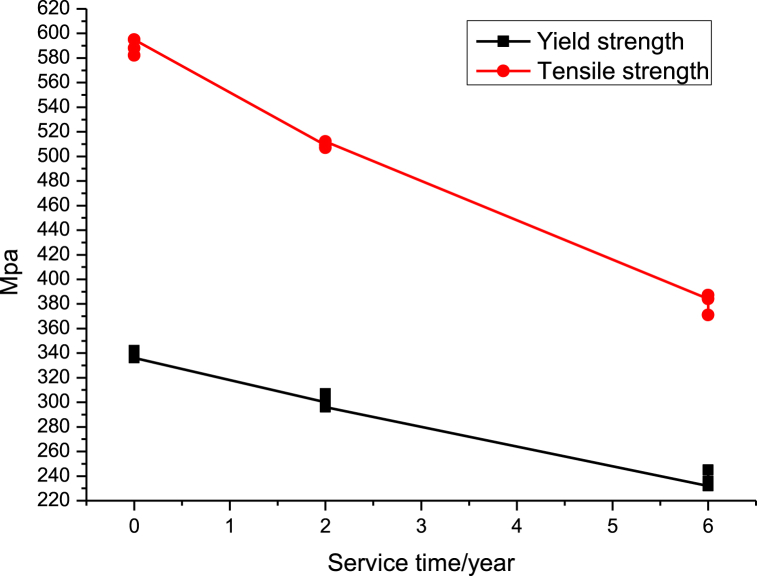
Variation curve of yield strength and tensile strength of furnace tube with service time.

**Fig. 11:**
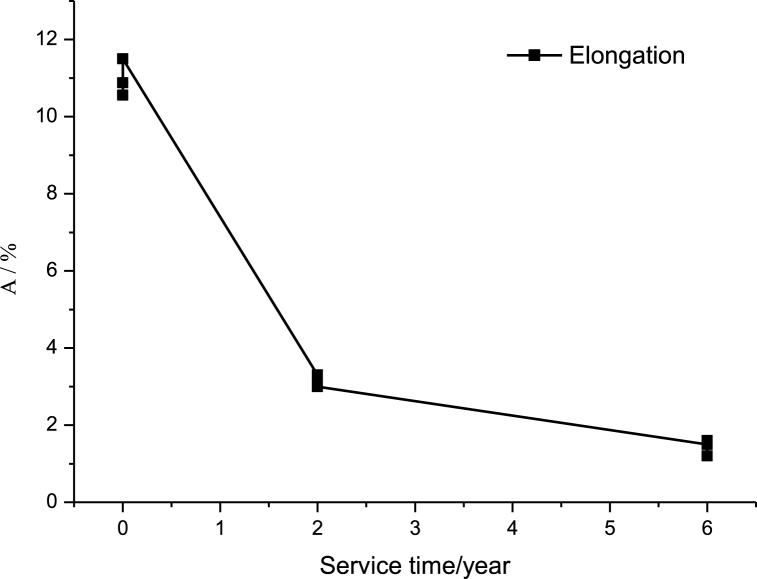
Variation curve of elongation after fracture with service time of cracking furnace tube.

We respectively from the unused furnace tube, the use of 2 years of furnace tube, and the use of 6 years of furnace tube on each of the three pieces of specimens were intercepted respectively numbered mechanical properties of the test, the specimen number, width, thickness, and the test temperature and the results of the test are shown in Table 3, Fig. 10, Fig. 11.

**Table 3:** Tensile test results for the furnace tube.

Service life	Specimen No.	Specimen size/mm		Yield strength $R_{p0.2}/\text{MPa}$	Tensile strength $R_{m}/\text{MPa}$	Elongation A/%	Test temperature°C
		Width	Thickness				
Unused	1	12.375	3.20	342.2	588	10.88	26
	2	12.400	3.12	338	582	10.56	26
	3	12.385	3.12	336	595	11.5	26
Service for 2 years	4	12.44	2.985	300	509	3.3	26
	5	12.47	2.998	307	507	3.1	26
	6	12.53	2.974	296	512	3.0	26
Service for 6 years	7	12.35	2.965	232	384	1.5	26
	8	12.37	2.977	236	387	1.2	26
	9	12.33	2.976	245	371	1.6	26

#### 3.2.2 Analysis of mechanical properties at room temperature

The above mechanical property tests show that the yield strength, tensile strength, and elongation of the stovepipe show a decreasing trend with the prolongation of the use time. As can be seen from Fig. 10, the yield strength and tensile strength decrease uniformly with the increase of service time, while the change curve of the elongation rate of the furnace tube with the increase of service time in Fig. 11 shows that the elongation rate does not decrease uniformly with the increase of service time, and there is a significant decrease in the first two years of the use of the furnace tube, while the later decreasing tendency is slowing down significantly, and the elongation rate of the tube used for 6 years is only 1.5 %. With the prolongation of service time, the tensile strength of the furnace pipe in service for 6 years decreased significantly; this may be due to the serious oxidation and carburization of the furnace tube material, as well as the prolongation of service time leading to the generation of creep holes. As a result, the plasticity, tensile strength, and yield strength of the tubes decreased.

### 3.3 Durability tests of the furnace tubes

**Fig. 12:**
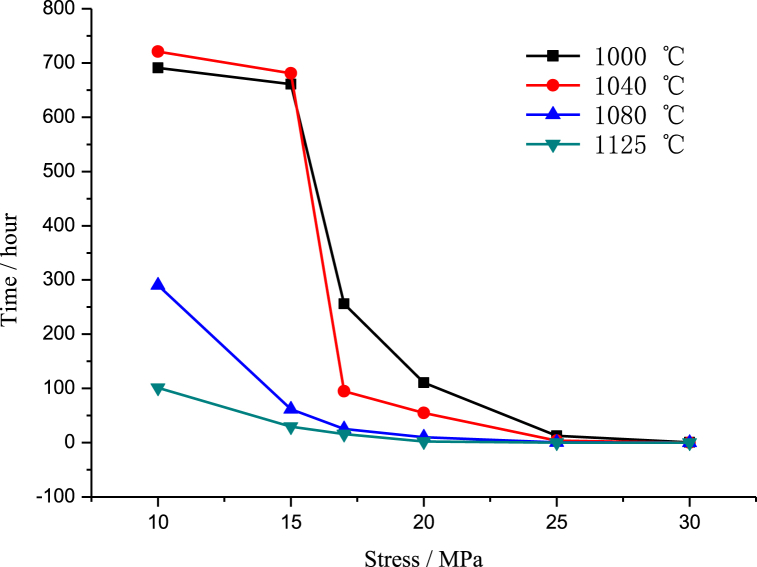
Durability test curves of unused furnace tubes at different temperatures and pressures.

We used six stresses (10 MPa, 15 MPa, 17 MPa, 20 MPa, 25 MPa, and 30 MPa) and four test temperatures (1000 °C, 1040 °C, 1080 °C and 1125 °C) to carry out the durability test in the standard atmospheric pressure environment. The test results are shown in Table 4, and the curves fitted with the test data are shown in Fig. 12.The durability test results for the unused furnace tube at different temperatures and pressures are listed in Table 4. The curves plotted using the data from Table 4 are shown in Fig. 12, from which it can be seen that the durability time of the cracker furnace tubes decreases with the increase of temperature and stress, in which there is a significant decrease in the durability time of the cracker furnace tubes when the temperature is more than 1040 °C and at a stress of 10 MPa. There is also a significant decrease in the durability time of the cracker tube after the stress exceeds 15 MPa.

**Table 4:** Durability test results for the unused furnace tube.

Stress/Temperature	1000 °C	1040 °C	1080 °C	1125 °C
10 MPa	691 h	721 h	290 h	101 h
15 MPa	661 h	681 h	61.5 h	29.3 h
17 MPa	256 h	94.8 h	25.5 h	15.6 h
20 MPa	110.5 h	54.8 h	10.2 h	2.2 h
25 MPa	12.5 h	3.5 h	0.5 h	0.05 h
30 MPa	0.5 h	0.2 h	0.01 h	0.01 h

**Fig. 13:**
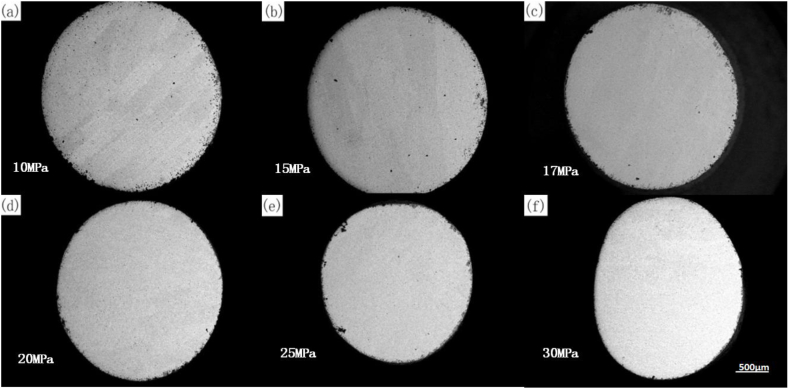
Macroscopic persistent organization at 1080 °C/different stresses ((a) 10 MPa; (b) 15 MPa; (c) 17 MPa; (d) 20 MPa; (e) 25 MPa; (f) 30 MPa).

**Fig. 14:**
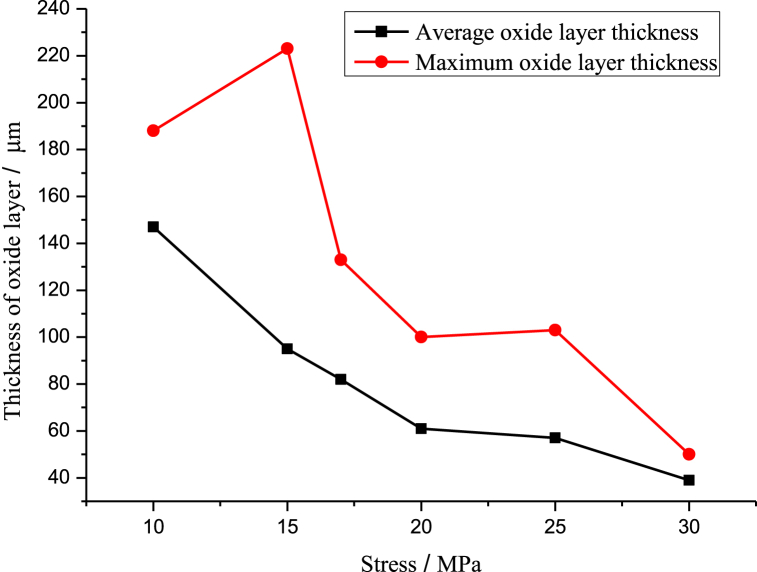
Variation curve of oxide layer thickness versus stress at 1080 °C.

**Fig. 15:**
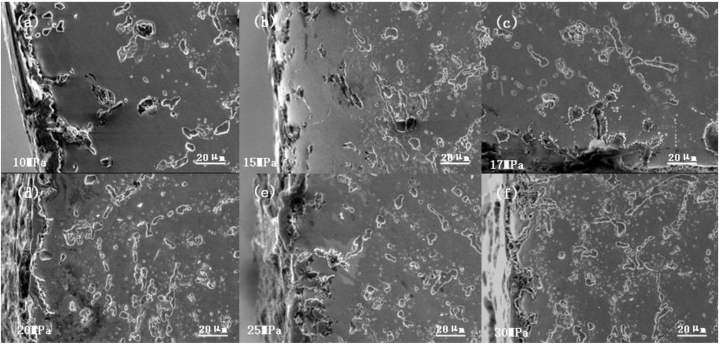
Shape of the oxide layer at 1080 °C under different stresses ((a) 10 MPa; (b) 15 MPa; (c) 17 MPa; (d) 20 MPa; (e) 25 MPa; (f) 30 MPa).

The durability test was conducted in an oxidizing environment. The surface oxide layer of the specimen exhibited different states under different durability conditions, as illustrated in Fig. 13 (a) - (f). Fig. 14 shows the variation curve of the oxide layer thickness versus stress at 1080 °C. At the same temperature, the average oxidation zone width decreased with the increase in stress. Furthermore, as illustrated in Fig. 15 (a) - (f), the endurance lifetime and fracture occurrence rate increased with the increase in stress, and thus no time was available for the development of the oxide zone.

To better study the relationship between oxide layer thickness and stress, we measured the thickness of the oxide layer at different stresses. The oxide layer was determined by SEM image characterization, direct measurement of the image, combined with the surface scanning of elements such as Si, Cr, and O to analyze the distribution of elemental content, and synthesized to determine the thickness of the oxide layer. Table 5 shows the maximum thickness, the minimum thickness, and the average thickness size of the oxide layer corresponding to different stress values at 1080 °C. Fig. 14 shows the curve of oxide thickness variation with stress at 1080 °C. At the same temperature, the average width of the oxidation zone decreased with the increase in stress because the endurance lifetime decreased and fracture occurrence rate increased with the increase in stress in the test environment; thus, the available time was not sufficient for the development of the oxide zone.

**Table 5:** Correspondence between stress and oxide thickness at 1080 °C.

Stress/Thickness of oxide layer	Maximum	Average	Minimum
10 MPa	188 μm	147 μm	106 μm
15 MPa	223 μm	95 μm	0 μm
17 MPa	133 μm	82 μm	31 μm
20 MPa	100 μm	61 μm	22 μm
25 MPa	103 μm	57 μm	11 μm
30 MPa	50 μm	39 μm	28 μm

Fig. 15 (a)–(f) shows the morphology of the oxidation zone of the furnace tube material under different stresses at 1080 °C. The surface oxide layer and the subsurface carbide-depleted zone were formed on the side of the specimen under the high-temperature oxidation environment, and the width of the carbide-depleted zone was closely related to the time of high-temperature oxidation. Thus, the persistent tissue under 10 MPa (Fig. 15 (a)) had the widest carbide-depleted zone, and the width of the depleted zone of the tissue displayed in Fig. 15 (f) was negligible because the fracture occurred in 80 min.

**Fig. 16:**
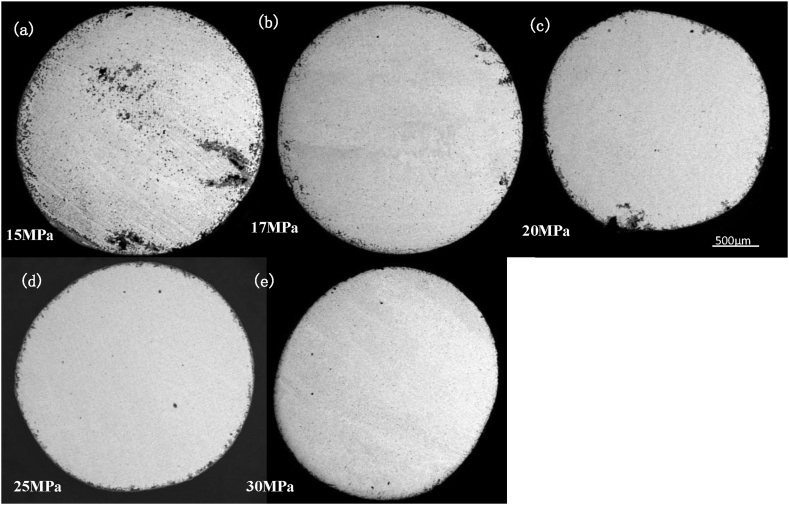
Macroscopic persistent organization at 1040 °C under different stresses ((a) 10 MPa; (b) 17 MPa; (c) 20 MPa; (d) 25 MPa; (e) 30 MPa).

Fig. 16 (a) - (e) shows the macroscopic persistent organization at 1040 °C under different stresses. Severe internal oxidation occurred in the specimen at 1040 °C under a stress of 15 MPa and even penetrated the core of the specimen, and the macroscopic persistent organization at 1080 °C under different stresses (Fig. 15 (a)–(f)) indicated that severe rupture damage of the oxide film occurred under low stress. Thus, stress can cause oxide film rupture damage, which is more evident at lower temperatures and under lower stresses, for longer test time, and stronger influence of internal oxidation corrosion.

Persistence experiments revealed that oxidation occurred in the furnace tube specimens at high temperatures, forming a dense protective oxide film, which generally results in increased resistance of the oxide to deformation compared with that of the base alloy. However, because the growth rate of the oxide film was considerably low and the film was thin, their influence on the creep rate of the specimen was weak. Furthermore, the oxide film is generally brittle; thus, when the creep rate is high, the deformation of the oxide film and the substrate does not coordinate to cause the cracking and peeling of the oxide film. The rupture of the oxide film can heal the crack through self-healing, and the newly formed oxide film cracks again after some time. This cracking–healing process occurs periodically and repeatedly, thereby accelerating the oxidation process.

## 4 Discussion

Long-term service of the furnace tube leads to substantial changes in the material organization, and these changes are correlated with endurance performance. For example, the organization of a furnace tube in service for 2 years mainly contains Nb_3_Ni_2_Si phase (i.e. G phase), austenite matrix, continuous massive M_23_C_6_ between primary dendrites, and secondary diffuse precipitation of M_23_C_6_ particles occurs within the crystal [25]. Furthermore, cavities are formed between primary dendrites or on the edges.

**Fig. 17:**
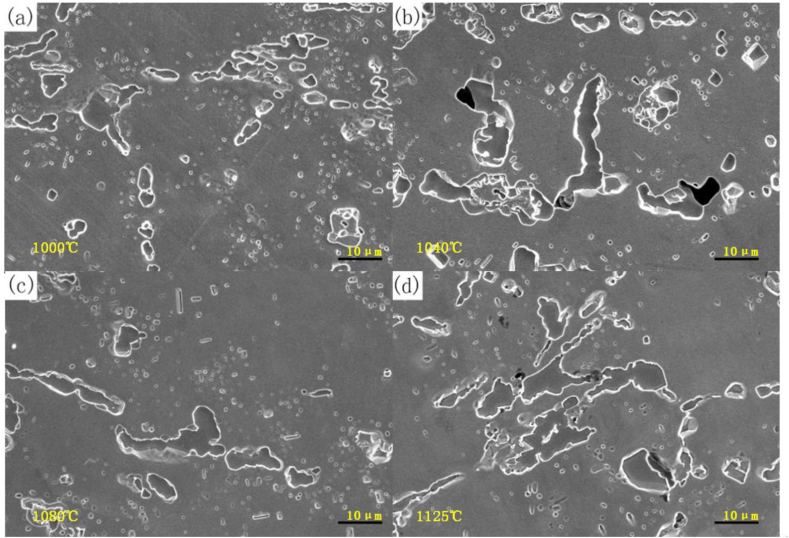
Persistent sample tissue under 20 MPa at different temperatures ((a) 1000 °C; (b) 1040 °C; (c) 1080 °C; (d) 1125 °C).

Fig. 17 (a) - (d) shows the metallographic organization of the persistent specimens after fracture at different temperatures under a stress of 20 MPa. The carbides in the tissue aggregated and increased in concentration, thereby weakening the effects of diffusion strengthening and grain boundary carbide strengthening and reducing the resistance to high-temperature deformation. In particular, the carbide coarsening was more evident under 20 MPa at 1125 °C, with a width of about 4.3 μm, and the concentration of secondary carbides also decreased. Such tissue degradation results in lower endurance lifetimes compared with other furnace tubes under the same pressure.

**Fig. 18:**
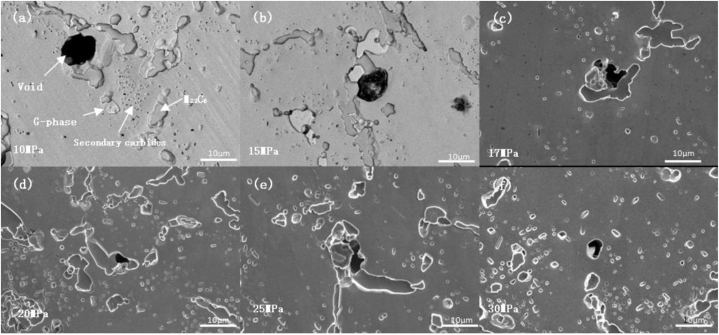
Creep voids in the persistent tissue at 1080 °C under different stresses ((a) 10 MPa; (b) 15 MPa; (c) 17 MPa; (d) 20 MPa; (e) 25 MPa; (f) 30 MPa).

The size and the number of the creep cavities increased with the coarsening of the carbide. The precipitate-free region around the carbide was easily deformed, and the thermal expansion coefficient of this region was different from that of the bulk carbide. This resulted in inconsistent deformation of the carbide and the matrix, resulting in an increase in stress at the phase boundary and the formation of voids. In addition, the voids were related to the diffusion of point defects on the grain boundary. During creep fracture, the stress concentration was smaller for particles of moderate size on the phase boundary, whereas the stress concentration was larger for both very small and larger particles. Fig. 18 (a) - (f) shows the creep cavity in the permanent structure at 1080 °C under different stresses.The observed organization indicated that voids were generated at the boundary of both primary M_23_C_6_ and G phase (Nb_3_Ni_2_Si), but the stress concentration was larger. Furthermore, voids were more likely to form because M_23_C_6_ was much larger than the G phase, whereas no voids were formed in the crystal or around the secondary carbide particles. The increase in cavity nucleation reduced the bearing area of the specimens, increased the actual stress, and gradually accelerated creep [[26], [27], [28]], thereby accelerating the growth of cavities and considerably decreasing the longevity of the furnace tube.

## 5 Conclusion

With the increase in service time, the width of an oxide layer and carbide-poor zone on the inner wall surface of the cracking furnace tube also increases correspondingly. 19.0 % is the critical concentration of carbide precipitation and decomposition in the carbide-poor zone of Cr35Ni45Nb alloy. When the concentration of Cr is higher than this value, carbides can exist stably without decomposition, which further leads to the formation of an internal carbide-rich zone. At the same time, with the increase in service time, the material properties of the furnace tube also deteriorate, and the yield strength, tensile strength, and elongation all show a downward trend, especially the elongation. The elongation of the furnace tube after six years of service is only 1.5 %, and the yield strength and tensile strength are less than 80 % of that of the new tube. The results of the furnace tube endurance test show that at the same temperature, the greater the stress, the shorter the endurance life of the furnace tube, and the smaller the average oxidation zone width because of the rapid fracture and insufficient time for oxidation.

Through the above conclusions, we can help to master the deterioration degree and remaining life of the in-service furnace tube, thus providing a basis for the decision-making of the service state and service performance of the furnace tube.

## CRediT authorship contribution statement

**Chenyang Du:** Writing – review & editing, Writing – original draft, Validation, Supervision, Project administration, Methodology, Investigation, Conceptualization. **Ce Song:** Writing – review & editing, Project administration, Investigation, Formal analysis. **Xiaowei Li:** Writing – review & editing, Validation, Supervision, Investigation. **Jun Yuan:** Validation, Supervision, Investigation, Data curation. **Chang Liu:** Writing – review & editing, Validation, Supervision, Investigation, Data curation. **Yanchao Xin:** Validation, Supervision, Investigation. **Jianyu Lu:** Validation, Supervision.

## Declaration of competing interest

The authors declare that they have no known competing financial interests or personal relationships that could have appeared to influence the work reported in this paper.
